# Supplementary material for: HIVprotI: an integrated web based platform for prediction and design of HIV proteins inhibitors
Source: J Cheminform. 2018 Mar 9;10:12. doi: 10.1186/s13321-018-0266-y (PMC5845081; doi:10.1186/s13321-018-0266-y)
Supplement: Supplementary file 2 — Additional file 2. Source code of HIVProtI web server. [file 13321_2018_266_MOESM2_ESM.zip › HIVprotI_Source-code/hivprotI/weka-3-6-6/doc/weka/core/EnvironmentHandler.html]

EnvironmentHandler


---


|  |  |  |
| --- | --- | --- |
| **Overview** \| **Package** \| **Class** \| **Tree** \| **Deprecated** \| **Index** \| **Help** \| **Weka's home** | |  |
| **PREV CLASS**   **NEXT CLASS** | **FRAMES**    **NO FRAMES**     **All Classes** |  |
| SUMMARY: NESTED \| FIELD \| CONSTR \| METHOD | DETAIL: FIELD \| CONSTR \| METHOD |  |


---


## weka.core Interface EnvironmentHandler

**All Known Implementing Classes:**: AbstractFileLoader, AbstractFileSaver, ArffLoader, ArffSaver, C45Loader, C45Saver, CSVLoader, CSVSaver, LibSVMLoader, LibSVMSaver, Loader, Saver, SerializedInstancesLoader, SerializedInstancesSaver, SerializedModelSaver, SVMLightLoader, SVMLightSaver, XRFFLoader, XRFFSaver

---

``` public interface EnvironmentHandler ```

Interface for something that can utilize environment
variables. NOTE: since environment variables should
be transient, the implementer needs to be careful
of state after de-serialization. Default system-wide
environment variables can be got via a call to
`weka.core.Environment.getSystemWide()`

**Version:**
:   $Revision: 5562 $

**Author:**
:   mhall (mhall{[at]}pentaho{[dot]}com)

---

| **Method Summary** | |
| --- | --- |
| `void` | `setEnvironment(Environment env)`             Set environment variables to use. |

**Method Detail**

### setEnvironment

```
void setEnvironment(Environment env)
```

:   Set environment variables to use.

    :   **Parameters:**: `env` - the environment variables to use


---


|  |  |  |
| --- | --- | --- |
| **Overview** \| **Package** \| **Class** \| **Tree** \| **Deprecated** \| **Index** \| **Help** \| **Weka's home** | |  |
| **PREV CLASS**   **NEXT CLASS** | **FRAMES**    **NO FRAMES**     **All Classes** |  |
| SUMMARY: NESTED \| FIELD \| CONSTR \| METHOD | DETAIL: FIELD \| CONSTR \| METHOD |  |


---
